# Effects of paternal exposure to cigarette smoke on sperm DNA methylation and long-term metabolic syndrome in offspring

**DOI:** 10.1186/s13072-022-00437-8

**Published:** 2022-01-21

**Authors:** Yunyun Liu, Shengzhu Chen, Dejian Pang, Jiayi Zhou, Xiuting Xu, Si Yang, Zhaofeng Huang, Bolan Yu

**Affiliations:** 1 Department of Obstetrics and Gynecology, BioResource Research Center, Key Laboratory for Major Obstetric Diseases of Guangdong Province, The Third Affiliated Hospital of Guangzhou Medical University, No. 63 Duobao Road, Liwan District, Guangzhou, 510150 Guangdong China; 2 Institute of Human Virology, Zhongshan School of Medicine, Sun Yat-sen University, Guangzhou, China

**Keywords:** Paternal exposure, DNA methylation, DLK1

## Abstract

**Background:**

Although paternal exposure to cigarette smoke may contribute to obesity and metabolic syndrome in offspring, the underlying mechanisms remain uncertain.

**Methods:**

In the present study, we analyzed the sperm DNA-methylation profiles in tobacco-smoking normozoospermic (SN) men, non-tobacco-smoking normozoospermic (N) men, and non-smoking oligoasthenozoospermic (OA) men. Using a mouse model, we also analyzed global methylation and differentially methylated regions (DMRs) of the DLK1 gene in paternal spermatozoa and the livers of progeny. In addition, we quantified DLK1 expression, executed an intra-peritoneal glucose tolerance test (IPGTT), measured serum metabolites, and analyzed liver lipid accumulation in the F1 offspring.

**Results:**

Global sperm DNA-methylation levels were significantly elevated (*p*  < 0.05) in the SN group, and the methylation patterns were different among N, SN, and OA groups. Importantly, the methylation level of the *DLK1* locus (cg11193865) was significantly elevated in the SN group compared to both N and OA groups (*p*  < 0.001). In the mouse model, the group exposed to cigarette smoke extract (CSE) exhibited a significantly higher global methylation DNA level in spermatozoa (*p*  < 0.001) and on the DMR sites of *Dlk1* in 10-week-old male offspring (*p*  < 0.05), with a significant increase in *Dlk1* expression in their livers (*p*  < 0.001). In addition, IPGTT and LDL levels were significantly altered (*p*  < 0.001), with elevated liver fat accumulation (*p*  < 0.05) in F1 offspring.

**Conclusion:**

Paternal exposure to cigarette smoke led to increased global methylation of sperm DNA and alterations to the DMR of the *DLK1* gene in the F1 generation, which may be inherited parentally and may perturb long-term metabolic function.

**Supplementary Information:**

The online version contains supplementary material available at 10.1186/s13072-022-00437-8.

## Introduction

There is growing evidence to support the hypothesis that paternal exposure to various contaminants, nutrients, and lifestyle-related conditions can influence an offspring’s future health—including increasing the risks for obesity, diabetes, and other chronic non-genetic diseases [1]. A report based on the Avon Longitudinal Study of Parents and Children cohort showed that a prepubertal exposure to father’s smoking was associated with son-specific obesity [2], and that the degree increased with age [3]. Another epidemiologic study revealed an association between paternal smoking and an offspring’s overweight/obesity that was more marked for boys [4]. Therefore, there is a likely association between father’s smoking and a son’s overweight/obesity. As smoking can lead to significantly increased production of reactive oxygen species (ROS) in sperm, and since ROS-induced oxidative stress may alter DNA methylation [5, 6], the changes in sperm DNA methylation constitute a plausible mechanism for the influence of father’s smoking on progeny; however, additional studies are needed for further clarification.

Imprinted genes play important roles in metabolic regulation, and associations between abnormalities in these genes and metabolic disease risk have been widely reported [7–9]. The paternal imprinting gene delta-like homolog 1 (DLK1) encodes a transmembrane protein that is related to adipocyte differentiation [10]. Clinical studies have demonstrated that DLK1 protein concentrations in serum are associated with obesity and glucose intolerance [11], and serum DLK1 levels have been found to increase with obesity [12]. DLK1 also exerts a direct negative impact on the systemic glucose homeostasis of obese patients [13]. In addition, DLK1-overexpressing mice were severely insulin resistant as represented by an insulin-stimulated diminution in glucose intake that resulted in increased blood glucose and inhibited fat production [14]. Therefore, the DLK1 gene is an important paternal imprinting gene that regulates offspring metabolism.

The expression of DLK1 is largely regulated by parent-specific DNA methylation, which includes primary and secondary differentially methylated regions (DMRs). The primary DMR is IG-DMR, which is located between DLK1 and Glt2, and secondary DMRs are located at DLK1/Glt2 promoters [15, 16]. DNA methylation of the IG-DMR is established during gametogenesis, and this occupies a critical position in marking parental origin, while secondary DMRs acquire DNA methylation after fertilization and may play a role in maintaining imprinting rather than being a major marker [15]. Evidence suggests that the primary DMRs are more genetically stable than secondary DMRs [17, 18]. Therefore, if the DMR of the DLK1 gene were to be interrupted paternally, this might affect the expression of DLK1 and long-term metabolism in offspring.

In the present study, we performed epigenetic analyses using a mouse model of paternal exposure to cigarette smoke extract (CSE) and also sperm DNA samples from tobacco-smoking men, and screened metabolically associated alterations in sperm DNA methylation in both taxa. The alterations we uncovered in DLK1 imprinting and expression were further verified in paternal sperm and liver cells of the F1 generation in mice. These results will help us understand how paternal exposure to cigarette smoke leads to impaired metabolism in F1 offspring, and whether such alterations are linked to transmission of cross-generational imprinting.

## Materials and methods

### Human subjects

This study was approved by the institutional review board of the Third Affiliated Hospital of Guangzhou Medical University. Normozoospermic and oligoasthenozoospermic men were enrolled according to their clinical semen examination record. Nonsmokers were randomly selected to match heavy smokers in clinical semen parameters and other individual characteristics. Analyses of semen parameters were as reported by clinics according to WHO standards. We enrolled 24 pairs of non-tobacco-smoking normozoospermic men and normozoospermic men who smoked heavily for comparisons of epigenetic indicators such as semen ROS, sperm histone-transition abnormalities, and global methylation levels in sperm DNA. The analyses of semen ROS and histone-transition abnormality were conducted according to procedures reported previously [19].

### Mouse breeding

For this study, we obtained experimental animal ethics approval from the Zhongshan School of Medicine of Sun Yat-sen University (Guangzhou, China). Briefly, 4-week-old specific-pathogen-free (SPF) C57BL/6N mice (30 males and 70 females) were purchased from the Vital River Laboratory Animal Technology Co., Ltd. (Beijing, China). The mice were fed under SPF conditions and exposed to an environment of 22 ± 1 °C, 60 ± 5% humidity, and twelve hours of lighting per day in the SPF Animal Facility of the Zhongshan School of Medicine of Sun Yat-sen University.

The males were allocated to an experimental group (designated CSE) and control group (designated CON). The CSE-group mice were fed with water containing 2 mg/mL of cigarette smoke extract (CSE) that was prepared and treated as described previously [20, 21]. When mice from both CSE and CON groups grew to be 10 weeks old, they were arranged to breed with 8-week-old females. The male and female offspring of the male CSE-group mice were denoted CSE-M and CSE-F, respectively, and the counterparts of the CON group were labeled as CON-M and CON-F, respectively. The pups were weaned from four weeks of age, and offspring of the CSE-M (*n*  = 20), CSE-F (*n*  = 25), CON-M (*n*  = 15), and CON-F (*n*  = 16) groups were fed normally to ten weeks of age.

### Methylation analysis

The gDNA was extracted from sperm cells and mouse livers using a MagPure Tissue DNA KF Kit (Magen, China) according to the manufacturer’s instructions and quantified with a Qubit4 Fluorometer (Life Technologies, USA). The global sperm DNA-methylation level was measured by a MethylFlashTM Methylated DNA Quantification Kit (Colorimetric) (EpiGentek, USA) following the manufacturer’s instructions.

For methylation-array sequencing, the gDNA (1 μg) was converted with bisulfite using the EZ-DNA Methylation Kit (Zymo Research, USA), and the bisulfite-converted DNA (400 ng) from men was isothermally amplified, enzymatically fragmented, purified, and hybridized on the Infinium 450K array (Illumina, USA) according to the manufacturer’s instructions. Data analysis was conducted by a commercial company (Lianchuan, Hangzhou, China). Briefly, the raw data was generated by GenomeStudio Methylation Analysis Algorithms, and further normalized after deviation correction and site filtering. The methylation levels of gene promoters, CpG island regions, and miRNA promoters were given in each sample, and differentially methylated sites were considered with the following screening criteria: *p*  < 0.05 and |Beta-Difference| ≥ 0.2. DNA samples from six normozoospermic (SN) men who were heavy smokers and six non-smoking normozoospermic (N) men, as well as six non-smoking oligoasthenozoospermic (OA) men designated as a disease control, were subjected to methylation array.

In addition, we analyzed the methylation levels of the IG-DMR at the DLK1/Glt2 locus from mouse livers and sperm cells by direct sequencing of PCR-amplified products after bisulfite conversion per previous studies [22, 23]. At first, primers were designed using Primer software (version 5.0, Canada) at the upstream and downstream positions of IG-DMR (Additional file 1: Table S1), and the initial template was methylation-converted gDNA. For the first round of amplification, the procedure was: 94 ℃ for 5 min, 20 cycles of 94 ℃ for 1 min, 54 ℃ for 30 s, 72 ℃ for 1 min, followed by 72 ℃ for 5 min using the IG-DMR region 1 primers in the Additional file 1: Table S1. After that, a 20-fold dilution of the PCR product was amplified with the following procedure: 94 ℃ for 5 min, 35 cycles of 94 ℃ for 1 min, 54 ℃ for 30 s,72 ℃ for 1 min, followed by 72 ℃ for 5 min using the IG-DMR region 2 primers in the Additional file 1: Table S1. Final PCR products were analyzed for cytosine and thymine content at CpG sites by Sanger sequencing (Igebio, Guangzhou, China).

### Quantitative RT-PCR

Total RNA from mouse livers was extracted using the RNeasy Plus Universal Mini Kit (Qiagen, Germany) as instructed by the manufacturer, and the concentration was measured with a Qubit4 Fluorometer (Life Technologies, USA). One microgram of RNA was used for cDNA synthesis using a PrimeScriptTM RT reagent Kit with gDNA Eraser (Takara, Japan) according to the manufacturer’s protocol, followed by real-time quantification using a GoTaq qPCR Master Mix (Promega, USA) on a QuantStudioTM 6 Flex System real-time PCR machine (Applied Biosystems, USA), following the manufacturer’s instructions. The PCR data are presented as relative expression using the 2^−ΔΔCt^ algorithm, and normalized to the geometric mean of the housekeeping control genes Gapdh and Actb. The gene primers are listed in Additional file 1: Table S1.

### Intra-peritoneal glucose tolerance testing and metabolic analyses

The progeny mice at 4 and 10 weeks of age were fasted for 12 h with free access to water only, and their tail-vein blood was collected to measure fasting blood sugar with an Omron glucometer. All mice were intraperitoneally injected with glucose at a dose of 2 g/kg, and their blood glucose levels were also measured at 30, 60, 90, and 120 min after injection. Orbital blood samples were collected from each of the mice by removing the eyeball and serum was separated. Triglycerides (TG), total cholesterol (T-CHO), high-density lipoprotein (HDL), low-density lipoprotein (LDL), and insulin (INS) were measured using commercially available kits (Jiancheng Biotech, China) according to the manufacturer’s protocols.

### Histologic staining

The hepatic and visceral adipose tissues were fixed in 10% formalin solution at 25 °C for 6 h and mounted onto poly-L-lysine-coated glass slides. The lipid droplets containing tissue were evaluated using hematoxylin and eosin (H&E) (Thermo Fisher Scientific, USA) staining, and the area of a single adipocyte was measured using Image J (National Institutes of Health, USA).

### Statistical analysis

We expressed the experimental results as means  ±  SEM, and statistically analyzed them using SPSS (version 25.0, USA) and GraphPad Prism (version 7.0, USA). Student’s *t* test was used for comparisons between two parametric groups, and the differences among multiple groups were analyzed by one-way ANOVA. A *p *value of  < 0.05 was considered significant.

## Results

### Sperm DNA-methylation patterns in smoking men

Our clinical data showed that all semen parameters such as the sperm density, semen volume, sperm motility, and sperm vitality were similar between normozoospermic men who smoked heavily and matched normozoospermic men who did not smoke (Table 1). However, normozoospermic men who were heavy smokers had significantly higher levels of seminal ROS, sperm histone-transition abnormalities, and global sperm DNA methylation, suggesting significant alterations in sperm DNA epigenetics (Table 1).

**Table 1:** Alterations in epigenetic indicators in smoking normozoospermic men

Parameters	N	SN
Cigarette pack years^a^	0	15.00 ± 2.64
Age (years)	32.54 ± 0.60	34.13 ± 0.99
Sperm density (× 10^6^ cells/mL)	99.23 ± 10.54	91.53 ± 10.91
Semen volume (mL)	2.77 ± 0.18	2.93 ± 0.27
Sperm vitality (%)	85.54 ± 1.99	86.04 ± 1.47
Sperm motility (%)	63.00 ± 2.38	57.96 ± 2.17
Semen ROS (U/L)	1515 ± 520	3807 ± 912*
Sperm histone-transition abnormality	9.98 ± 0.88	12.92 ± 1.03*
Methylation level of sperm DNA (%)	1.94 ± 0.16	2.44 ± 0.16*

Data are presented as means  ±  SEM

*N* normozoospermic men who did not smoke; *SN* normozoospermic men who smoke heavily. ^a^Cigarette pack years was calculated by multiplying smoking dose (packs per day) by duration (years of smoking)

^*^p  < 0.05 compared to the N group

**Fig. 1:**
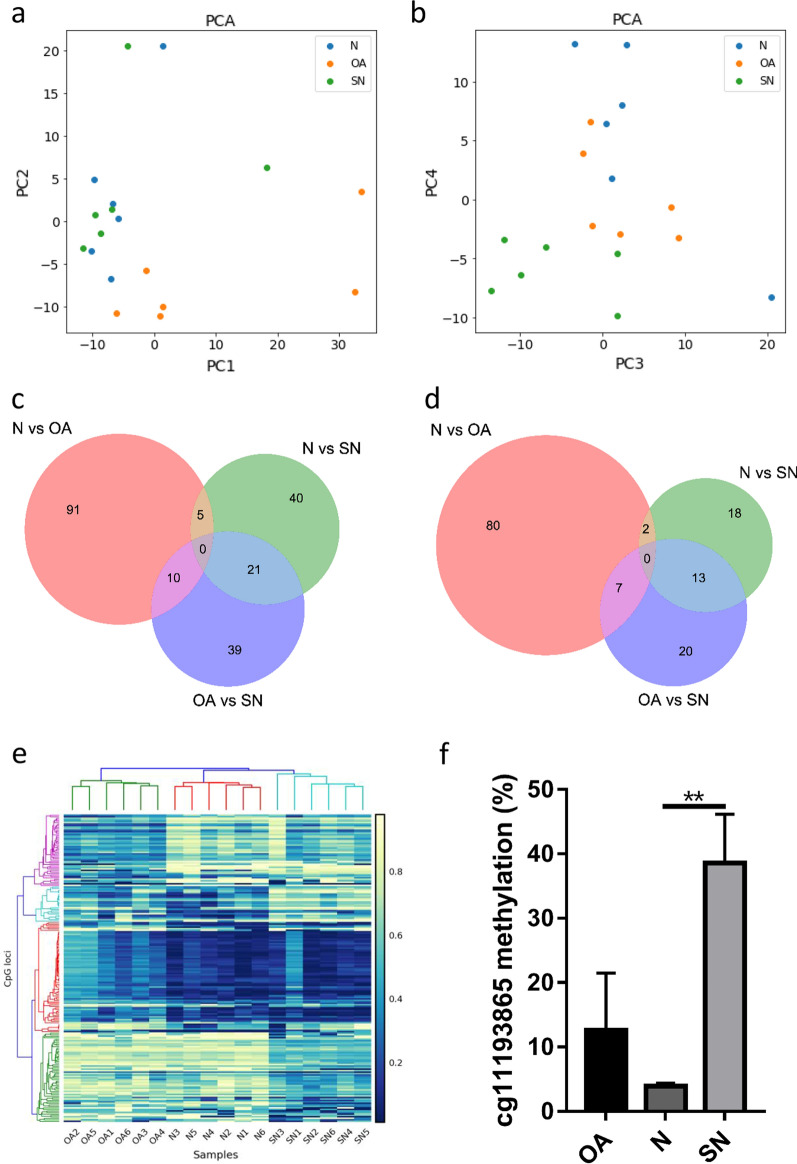
Analyses of human sperm DNA-methylation patterns in different groups. Principal component analysis (PCA) of Illumina 450K methylation-array beta values showed that OA separated from N and SN in the PC1 and PC2 planes (**a**), and that N separated from SN in the PC3 and PC4 planes (**b**). Venn diagram of significantly differentiated loci among groups in the number of CpG loci (**c**) and genes (**d**). **e** Hierarchical clustering of spermatozoal DNA-methylation patterns with differentiated loci among groups. **f** The methylation level on the cg11193865 site of the *DLK1* gene. *N* normozoospermic men without smoking; *SN* normozoospermic men with heavy smoking; *OA* oligoasthenozoospermic patients without smoking. **p  < 0.01 compared to the N group

Methylation array analysis revealed significant differences in methylation patterns among groups of non-smoking oligoasthenozoospermic men (OA), non-smoking normozoospermic men (N), and normozoospermic men who smoked heavily (SN) (Fig. 1). The principal component analysis (PCA) showed that the global methylation profiles of N, SN and OA subjects were highly heterogeneous as they had extensive variation even within groups (Fig. 1a, b). However, OA still had an ideal separation effect on N and SN at the PC1 and PC2 planes (Fig. 1a), and N had a good separation effect on SN at the PC3 and PC4 planes (Fig. 1b). Cluster analysis indicated that N, SN, and OA groups exhibited disparate sperm DNA-methylation profiles, with uniquely altered genes or a CpG island in each group (Fig. 1c–e). Importantly, array results suggested alterations in the methylation of paternal imprinting genes, as the cg11193865 site in the *DLK1* gene displayed a significantly higher methylation level in the SN group relative to either the N or OA group (*p * < 0.01, Fig. 1f).

### Methylation patterns in paternal sperm and progeny livers

**Fig. 2:**
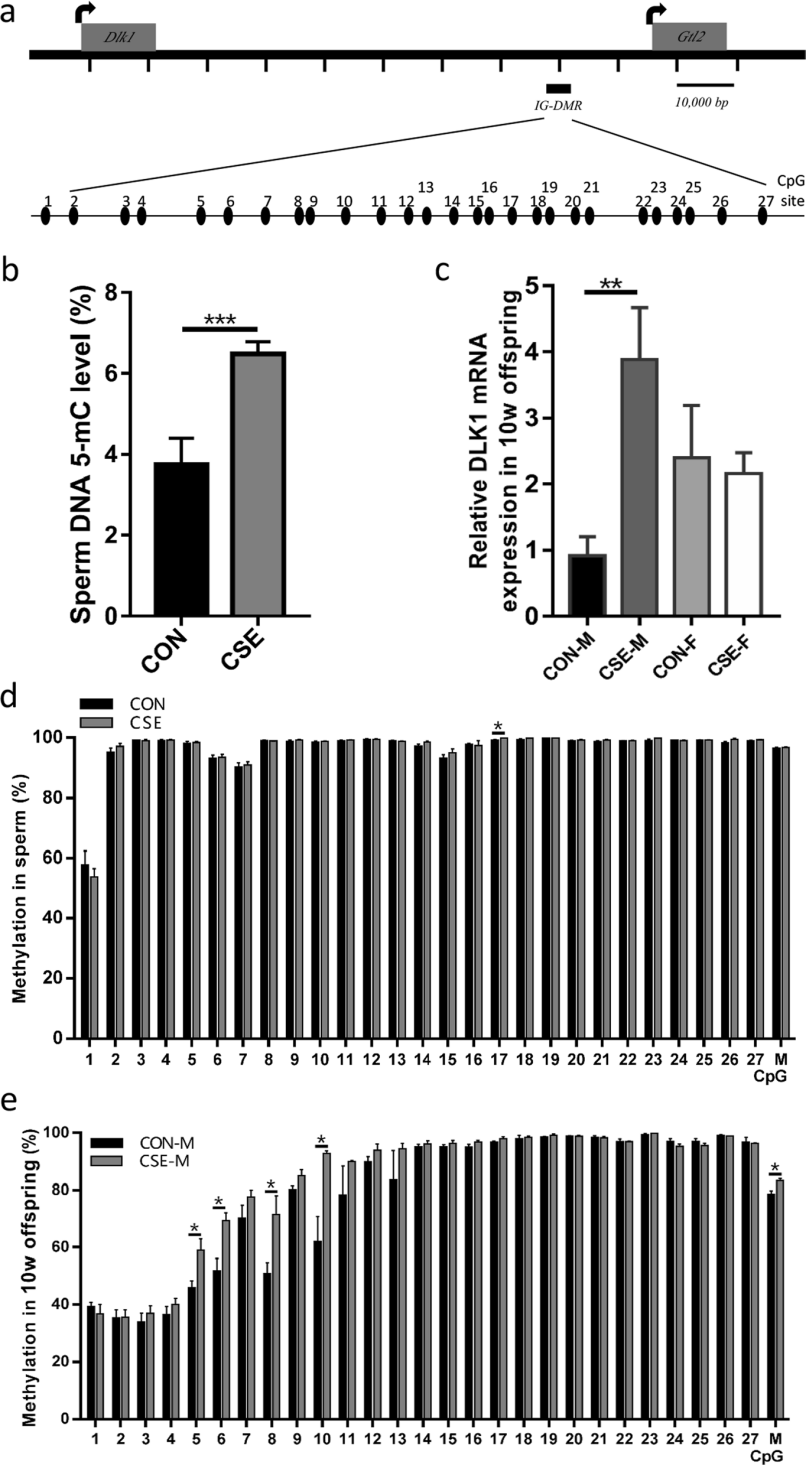
Methylation and *Dlk1* expression levels in paternal sperm and the F1 generation of the mouse model. **a** Model of the CpG site in the IG-DMR at the *Dlk1*/*Gtl2* locus in mouse. **b** Global DNA-methylation level (5-mC) in paternal sperm cells. **c** Expression level of *Dlk1* in liver from 10-week-old F1 offspring. **d** Methylation levels of the IG-DMR in paternal sperm cells. **e** Methylation levels of IG-DMR in F2 liver cells. IG-DMR, intergenic differentially methylated region. *M* mean of 27 CpG sites. *CON-M* and *CON-F* male and female offspring of the CSE group; *CSE-M* and *CSE-F* male and female offspring of the CON group. *p  < 0.05.**p  < 0.01.***p  < 0.001 compared to the CON group

In the mouse model, the CSE group exhibited a significantly higher global DNA-methylation level compared to the CON group (at the 5-mC nucleotide) (*p*  < 0.001, Fig. 2b). As methylation levels are extremely high in the IG-DMR, these alterations are difficult to be detected in sperm cells. However, the methylation level of the 17th site (Fig. 2a) of the IG-DMR at the *Dlk1*/*Gtl2* locus was significantly higher in the CSE group than that in the CON group (*p*  < 0.05, Fig. 2d), suggesting altered methylation in paternal sperm.

In the liver tissue of the F1 generation, there was a significantly higher methylation level of the IG-DMR at the *Dlk1*/*Gtl2* locus in the CSE group compared to the CON group, e.g., at sites 5, 6, 8, and 10 of the IG-DMR of 10-week-old male progeny (*p*  < 0.05, Fig. 2e). We also observed a significantly higher mean methylation level at the IG-DMR of the CSE exposure group in four-week-old male offspring compared with the CON group (unpublished data).

### The expression of *Dlk1* in progeny livers

In the 4-week-old pups, the expression level of *Dlk1* in the liver was not statistically different between CSE and CON groups, although the CSE group showed a tendency for higher expression levels, especially in the male offspring (Fig. 2c). In the 10-week-old male offspring, there were statistically significant differences in the expression levels of *Dlk1* in livers between the two groups (*p*  < 0.01, Fig. 2c), but there was no difference in expression in the 10-week-old female pups (Fig. 2c).

### Metabolic alterations in progeny mice

**Fig. 3:**
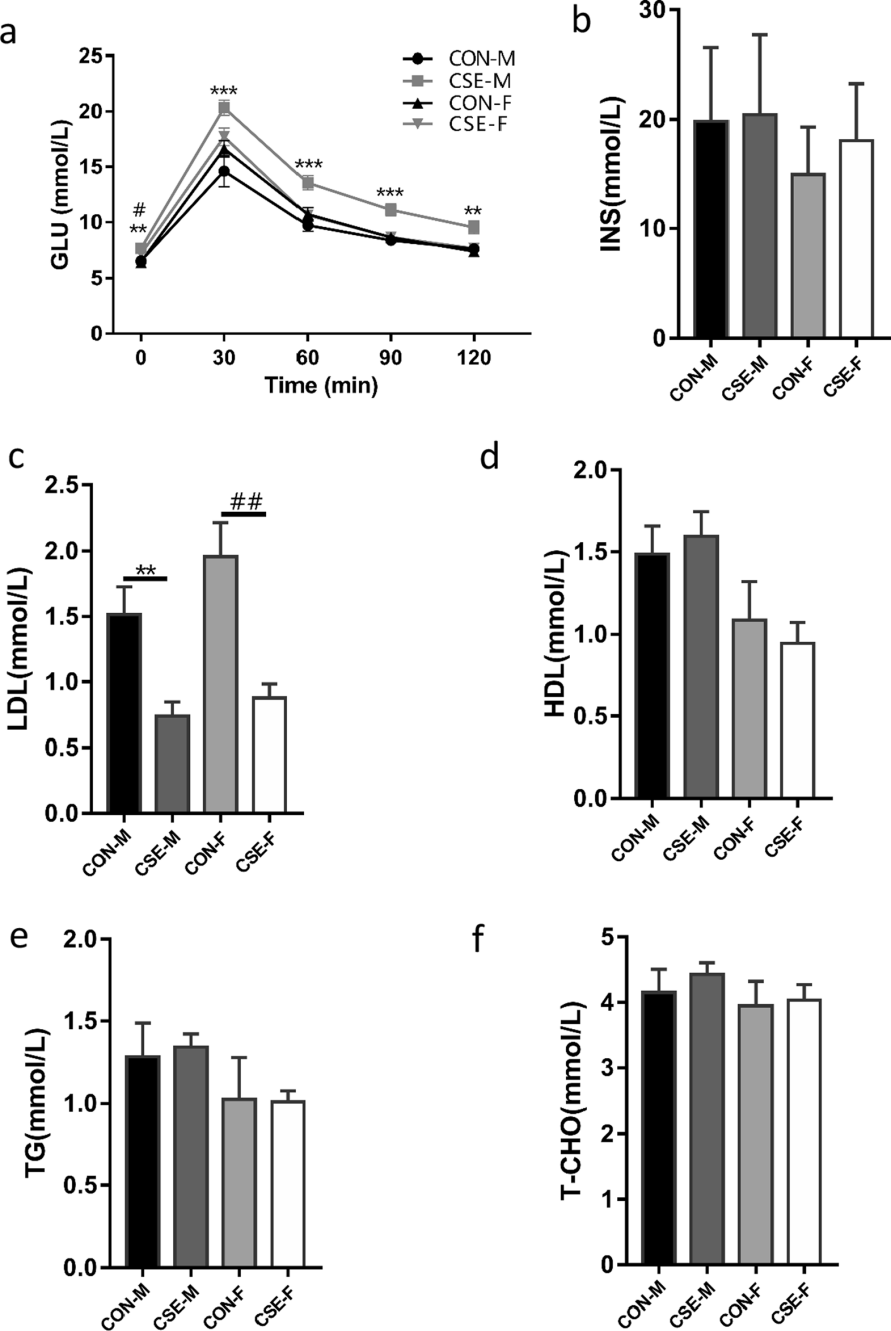
Metabolic alterations in 10-week-old offspring in different groups. **a** Blood glucose curves in offspring evaluated by intra-peritoneal glucose tolerance test (IPGTT) and **b** insulin (INS) in offspring of different groups. Serum levels of low-density lipoprotein (LDL, **c**), high-density lipoprotein (HDL, **d**), triglyceride (TG, **e**), and total cholesterol (T-CHO, **f**) in offspring of different groups. *CON-M* and *CON-F* male and female offspring in the CSE group; *CSE-M* and *CSE-F* male and female offspring in the CON group. ^**#**^*p*  < 0.05, between CON-F and CSE-F groups; ^**##**^*p*  < 0.01, between CON-F and CSE-F groups; **p  < 0.01, between CON-M and CSE-M groups; ***p  < 0.001, between CON-M and CSE-M groups

Although we noted statistical differences in four-week-old males in serum GLU at 60 min of the IPGTT test, there were no differences in LDL, T-CHO, TG, HDL, or INS levels between the CSE group and the CON group (unpublished data). However, the level of serum GLU showed significant differences for 10-week-old males at all times in the IPGTT between the two groups (*p*  < 0.05, Fig. 3a), and the LDL level was significantly lower in the CSE group relative to the CON group (*p*  < 0.01, Fig. 3c). The 10-week-old females of the CSE group also had significantly decreased LDL but not GLU levels compared to the controls (Fig. 3a, c). There were no differences in T-CHO, TG, HDL, or INS levels between groups (Fig. 3b, d–f).

We did not observe any significant weight gain for offspring from the CSE group. However, if all groups of mice were fed a high-fat diet, the weight gain for male offspring from the CES group was the highest among all groups, indicating that murine F1 offspring with paternal exposure to CSE was more likely to be affected by a high-fat diet (unpublished data).

### Analysis of lipid accumulation in offspring mice

**Fig. 4:**
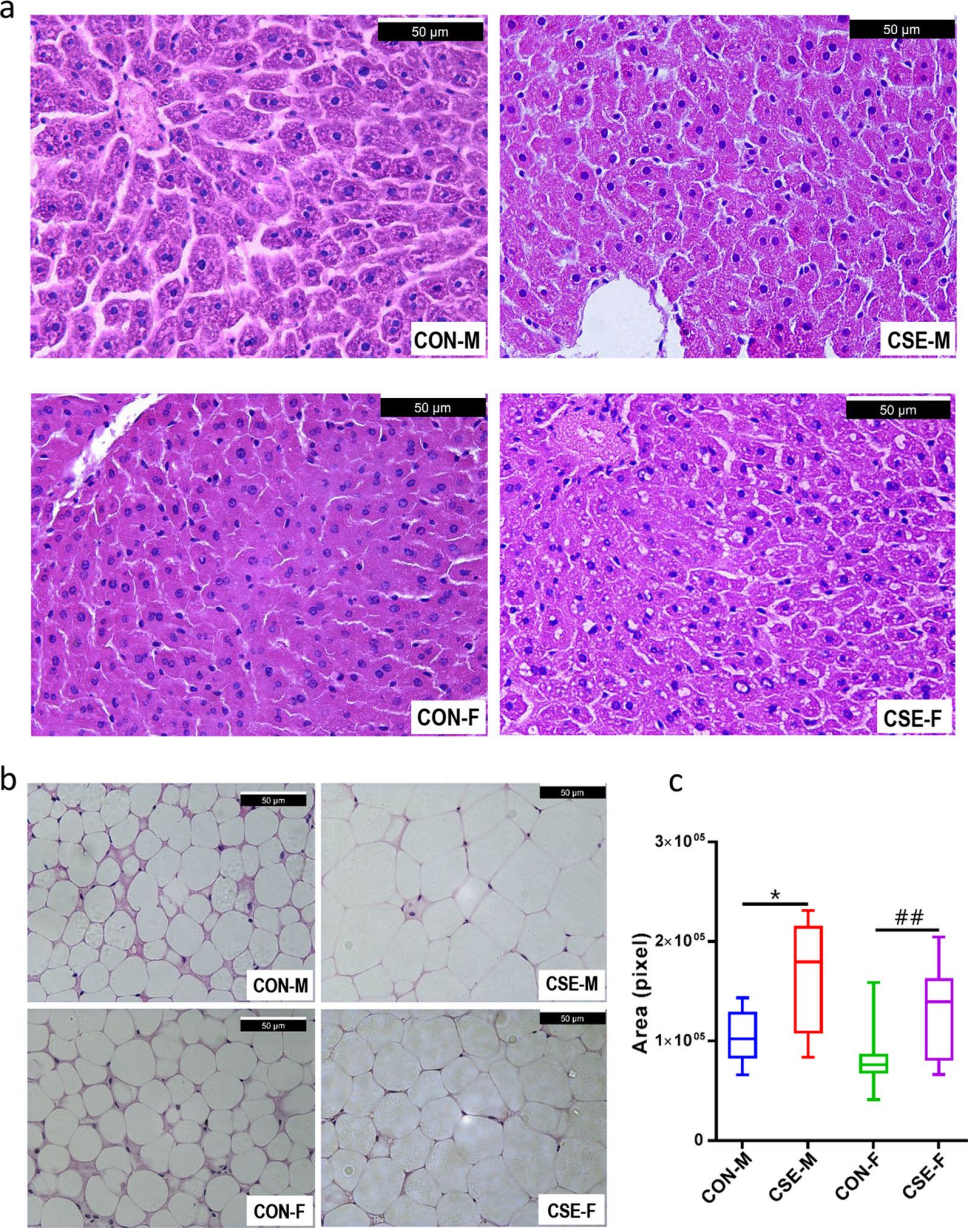
H&E staining of liver and visceral adipose tissue from 10-week-old offspring in different groups. **a** H&E staining of liver tissue. **b** H&E staining of visceral adipose tissue. **c** Area of adipose cells in different groups. *p  < 0.05, between CON-M and CSE-M groups; ^**##**^*p*  < 0.01, between CON-F and CSE-F groups

The H&E staining of liver tissue showed that there were more lipid droplets in the CSE group compared to the CON group of the 10-week-old offspring mice (Fig. 4a). In addition, the cross-sectional areas of visceral adipose tissue cells stained with H&E in the CSE group were larger than in the CON group (Fig. 4b), and we noted a statistically significant difference in the mean area of adipocytes (*p*  < 0.05, Fig. 4c). In addition, the results supported that the females were likely to develop pathological conditions in livers (Fig. 4a) and had a more significant increase in areas of visceral adipose tissue cells (Fig. 4c).

## Discussion

### Exposure to cigarette smoke causes abnormal sperm DNA-methylation profiles

Previous studies have shown that the mechanisms underlying transgenerational (paternity) effects include abnormal DNA methylation [24], histone expression imbalance [25], and non-coding RNA expression variations [26]. As reactive oxygen species (ROS) produced by cigarette smoke can change the epigenetic characteristics of sperm by interfering with the activities of enzymes such as DNMT, such types of epigenetic changes can be transferred to the next generation and contribute to phenotypic changes and the development of disease in offspring [27]. We also previously demonstrated that smoking significantly augmented ROS levels in male semen and attenuated the rate of sperm histone transition [19], and that the global DNA-methylation level of high-quality spermatozoa was significantly lower than that of low-quality sperm [28]. These studies indicated that paternal exposure to cigarette smoke altered important epigenetic characteristics in sperm DNA.

With the current study, we confirmed that smoking significantly elevated ROS levels and sperm histone-transition abnormalities, along with increased DNA-methylation levels in sperm (Table 1; Fig. 1). Using the mouse model, we herein observed that global DNA-methylation levels of the CSE group were also significantly higher than in the control group (Fig. 2). These data are consistent with recent reports that the global DNA-methylation levels of non-smokers are significantly lower than that of smokers, and that DNA methylation is significantly correlated with sperm parameters and non-condensed sperm chromatin [29].

Our data revealed that the global methylation profiles of non-smoking oligoasthenozoospermic men, smoking normozoospermic men, and non-smoking normozoospermic men were highly heterogeneous, but methylation patterns were significantly different among the three groups (Fig. 1). Jenkins et al. previously investigated the impact of exposure to cigarette smoke on sperm DNA-methylation patterns and reported consistently altered methylation at specific CpGs or within specific genomic regions, identifying 141 significantly differentially methylated CpGs associated with smoking [30]. We demonstrated in the current study that even in normozoospermic men, smoking affected the epigenetic profiles of sperm cells. After the addition of oligoasthenozoospermic men as another control, the N, SN, and OA groups manifested varying characteristics of sperm DNA-methylation profiles; however, the specific CpGs and genes we identified were not consistent with those reported by Jenkins [30]. The disparities between our studies were likely due to the smokers in their study displaying lower semen parameters than the controls, whereas in our study we compared smoking and non-smoking groups who exhibited similar clinical semen parameters. Therefore, the data supported our hypothesis that smoking and infertility affect global sperm DNA-methylation profiles differentially, and that smoking-associated DNA modifications constitute an important source of epigenetic alterations in sperm DNA—even without influencing clinical semen parameters.

### Paternal exposure to cigarette smoke leads to altered methylation of the DLK1 DMR in parental and F1 generations

Perturbation of sperm DNA methylation in fathers exposed to cigarette smoke may be transmitted across generations and confers health risks to their offspring. For example, in animal models alcohol and a high-fat diet can transform the DNA-methylation profile of male parents and pass it on to offspring [31, 32]. Our results showed that the DNA-methylation level of the DLK1 locus (cg11193865) in the men’s sperm was significantly elevated in normozoospermic men who were heavy smokers compared with oligoasthenozoospermic patients and non-smoking normozoospermic men. We postulate that this phenomenon may then disturb paternal imprinting status and further affect the expressive regulation of DLK1 in their children.

As it is difficult to study DLK1-methylation patterns in human F1 generations, we evaluated these pattern variations in the mouse model. The mouse paternal imprinting gene DLK1 principally exhibits three DMRs; i.e., DLK1-DMR, IG-DMR located 15 kB upstream of the Gtl2 gene, and GTL2-DMR across from the Gtl2 promoter and the first exon [33]. Our mouse data showed that the locus of the DLK1 IG-DMR was altered in the paternal sperm, which is consistent with our results in human sperm where DLK1 DMR methylation was interrupted (Fig. 2). Therefore, paternal exposure to cigarette smoke may not only increase the overall methylation level of sperm DNA but also modify the methylation of regulatory regions of the paternally imprinted gene DLK1.

Our data additionally revealed that in combination with altered DLK1 IG-DMR methylation, DLK1 expression in liver tissue from 10-week-old male progeny was significantly elevated (Fig. 2). Numerous studies have shown that the regulation of these DMRs by methylation plays a key role in the expression of the DLK1 gene [18, 34], and previous reports showed that the IG-DMR is inherited by the offspring from the father, and that the IG-DMR methylation in fathers may affect the expression of DLK1 in offspring [34] and be passed on to the next generation [35]. However, based on current data, the methylation pattern of DLK1 in the offspring livers was not directly inherited from their fathers. Considering that the methylation pattern can be established after embryo reprogramming and change along with life span, a conclusion of a paternal methylation pattern cannot be made yet.

### Paternal exposure to cigarette smoke leads to abnormal metabolic function in F1 offspring

Our analyses detected significantly elevated glucose levels and reduced LDL concentrations in 10-week-old offspring from CSE groups, and the enlargement of fat cells and increase in fat droplets in their livers—indicating relatively long-term effects of paternal exposure to cigarette smoke on progeny metabolism (Fig. 3). Recent studies have shown that serum DLK1 plays an important role in the metabolism of glycolipids throughout the body. Glucose transporters such as GLUT4 regulate glucose metabolism [13, 36], and serum DLK1 levels were significantly elevated commensurate with diminished DLK1 levels after weight-loss surgery in obese individuals [12]. In pregnant women, the DLK1 content in peripheral serum was also positively correlated with the levels of glycolipid metabolites such as TG, cholesterol, and LDL [37]. Therefore, the *DLK1* gene is an important regulatory factor in glycolipid metabolism, and its abnormal expression and regulation can lead to altered lipid metabolism. Since we observed alterations in *DLK1* DMR methylation and DLK1 expression in offspring after paternal exposure to CSE, DLK1 may provide an important clue to obesity induced by paternal exposure to cigarette smoke.

Our data also addressed significantly abnormal glucose metabolism and associated alterations in DLK1 expression and methylation in 10-week-old male—but not female—progeny of the CSE groups, indicating a sex-specific effect (Fig. 3). These results are consistent with epidemiologic observations that paternal cigarette exposure may be associated with increased risk for obesity in boys solely [2–4]. As the DMR region of DLK1 is tissue-specific—showing different DMR-methylation profiles in placenta, liver, and tumor tissues [38]—it may be also regulated differentially in the two sexes. In addition, females were more likely to develop pathological conditions in their livers (Fig. 4) with unknown reasons. Interestingly, it was found that a locus (cg09993711) on body region of estrogen receptor ESRB displayed significant higher methylation levels in sperm DNA from smoking men. As the estrogen signaling pathway plays a critical role in metabolism regulation in females, this may be an important clue for future investigation on the observed sex specificity.

## Conclusions

In summary, using both human sperm samples and a mouse model, we found that exposure to cigarette smoke significantly increased global methylation and altered DMR methylation of the DLK1 gene in sperm cells. Paternal exposure to cigarette smoke also caused significantly abnormal glucose and lipid metabolism and elevated liver fat accumulation in 10-week-old male offspring. The DMR-methylation pattern of DLK1 was significantly altered, as was the expression of DLK1 in the livers of male progeny. We therefore showed that paternal exposure to cigarette smoke led to altered DMR methylation of the paternally imprinted DLK1 gene, which was transmitted cross-generationally and induced abnormal metabolism in offspring. This study is expected to assist us in understanding the mechanisms observing obesity that are triggered by the father’s cigarette smoking as reported by epidemiology, and to provide new clues for metabolic diseases associated with intergenerational inheritance.

## Supplementary Information

**Additional file 1: Table S1.** Primers for IG-DMR amplification and RT-PCR experiment.

## Data Availability

Deidentified participant data are available upon reasonable requisition.
