# Supplementary material for: Effects of paternal exposure to cigarette smoke on sperm DNA methylation and long-term metabolic syndrome in offspring
Source: Epigenetics Chromatin. 2022 Jan 21;15:3. doi: 10.1186/s13072-022-00437-8 (PMC8780762; doi:10.1186/s13072-022-00437-8)
Supplement: Supplementary file 1 — Additional file 1: Table S1. Primers for IG-DMR amplification and RT-PCR experiment. [file 13072_2022_437_MOESM1_ESM.docx]

**Supplementary Table**

**Table S1**. Primers

|  | Forward primer | | Reverse primer |
| --- | --- | --- | --- |
| IG-DMR region 1 | | GTATGTGTATAGAGATATGTTTATATGGTA | CTTCCCTCACTCCAAAAATTAAAA |
| IG-DMR region 2 | | GTGTTAAGGTATATTATGTTAGTGTTAGGA | CTTCCCTCACTCCAAAAATTAAAA |
| GAPDH | | TGGTGAAGGTCGGTGTGAAC | CCATGTAGTTGAGGTCAATGAAGG |
| β-Actin | | GGCACCACACCTTCTACAATG | GGGGTGTTGAAGGTCTCAAAC |
| DLK1 | | TGCGCCAACAATGGAACTTG | TCTCGCATGGGTTAGGGGTA |

Primers for IG-DMR amplification and RT-PCR experiment.
